# Tokenization and deep learning architectures in genomics: A comprehensive review

**DOI:** 10.1016/j.csbj.2025.07.038

**Published:** 2025-07-28

**Authors:** Conrad Testagrose, Christina Boucher

**Affiliations:** Department of Computer and Information Science and Engineering, University of Florida, Gainesville, FL, United States

**Keywords:** Deep learning, Large language models, Tokenization, Genomics, DNA sequencing

## Abstract

The development of modern DNA sequencing technologies has resulted in the rapid growth of genomic data. Alongside the collection of this data, there is an increasing need for the development of modern computational tools leveraging this data for tasks including but not limited to antimicrobial resistance and gene annotation. Current deep learning architectures and tokenization techniques have been explored for the extraction of meaningful underlying information contained within this sequencing data. We aim to survey current and foundational literature surrounding the area of deep learning architectures and tokenization techniques in the field of genomics. Our survey of the literature outlines that significant work remains in developing efficient tokenization techniques that can capture or model underlying motifs within DNA sequences. While deep learning models have become more efficient, many current tokenization methods either reduce scalability through naive sequence representation, incorrectly model motifs or are borrowed directly from NLP tasks for use with biological sequences. Current and future model architectures should seek to implement and support more advanced, and biologically relevant, tokenization techniques to more effectively model the underlying information in biological sequencing data.

## Graphical abstract

**Figure:**
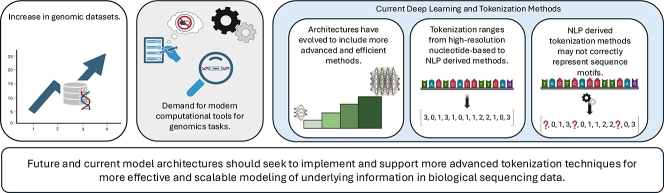

## 1 Introduction

The rapid growth of biological sequencing data has necessitated the development of accurate, high-throughput computational models for analyzing these data. Traditionally, biological sequence analysis tasks were carried out using statistical models, such as Hidden Markov Models (HMMs) [63], [79]. HMMs have seen application in gene prediction [9], [48], sequence alignment [51], prediction of the protein secondary structure [76], base calling [43], among other tasks. Although these approaches are effective, they often require significant domain expertise due to manual feature design or alignment tuning [61].

Expanding upon these statistical models, traditional machine learning (ML) algorithms have been applied to tasks related to biological sequence analysis. Methodologies utilizing ML have traditionally used logistic regression [69], decision trees [64] or random forests [15], [69], and support vector machines [24]. When using traditional ML algorithms in genomics, biological sequences are typically converted to feature vectors (e.g., *k*-mer frequencies, physicochemical properties, or position-specific scores) prior to training. These methods have been shown to achieve acceptable results; however, their performance is highly dependent on the quality of the feature engineering and extraction performed. Additionally, these traditional ML algorithms struggle to capture long-range dependencies native to biological sequencing data.

To overcome these limitations, deep learning has emerged as a powerful tool in the field of genomics. For example, convolutional neural networks (CNNs) have been successfully used for motif prediction [2], forecasting the effects of genomic variants on chromatin features [82], and classifying the functional activity of DNA sequences [34]. Their ability to capture complex patterns often reduces the need for extensive manual feature extraction and engineering. Additionally, the use of architectures like Long Short-Term Memory (LSTM) networks, Recurrent Neural Networks (RNNs), CNNs, and more recently transformer-based models [20], [56], [72] has enabled the capture of both local and global contextual information within genomic sequences.

More recently, Large Language Models (LLMs) based on the transformer architecture [72], such as BERT [20] and OpenAI's GPT [56], have demonstrated remarkable success in various NLP tasks such as text classification [20] and text generation [56]. This success in NLP has inspired the adaptation of LLMs to other domains, including bioinformatics. The analysis of biological sequences, such as DNA, RNA, or protein sequences, presents unique challenges. Unlike natural human language, biological sequences are non-ambiguous, lack delimiters or punctuation, and often span lengths far beyond the typical text corpora; this places heightened importance on the design of appropriate tokenization and architectural design methodologies critical to the success of these models in bioinformatics.

Therefore, this review provides a comprehensive survey of deep learning models in genomics and the sequence representation (tokenization) strategies essential for their success. We will examine the biological significance, advantages, and limitations of these pivotal applications. Additionally, we highlight emerging architectures and future directions that can help ensure that future models can overcome current limitations in scalability, computational cost, and biological interpretability.

## 2 Tokenization and deep learning architectures in genomics

**Fig. 1:**
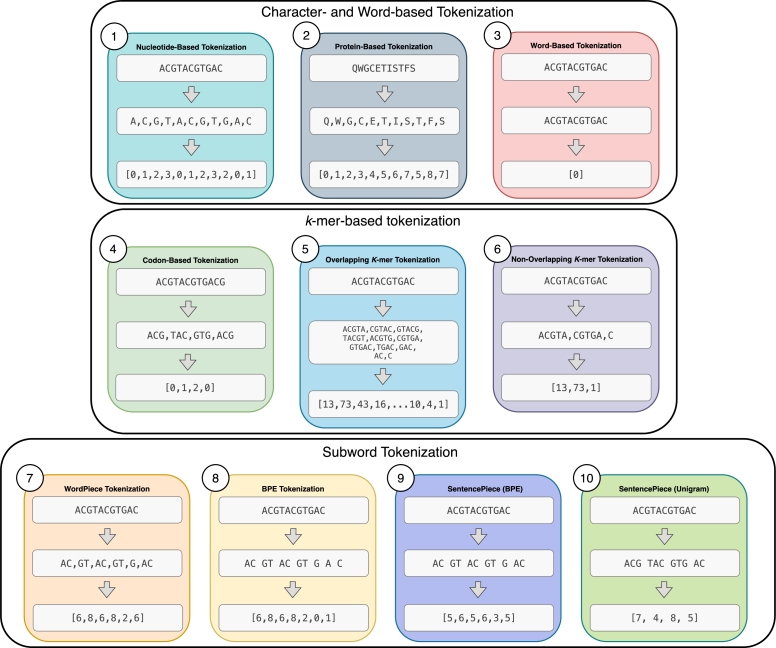
Examples of commonly used tokenization techniques on a sample of DNA sequence. 1-3: Nucleotide- or Protein-based tokenization results in each individual nucleotide represented as a single token, while Word-based Tokenization results in the whole sequence being represented as a single token. 4-6: Codon-based Tokenization and Non-overlapping *k*-mer Tokenization result in *k*-mers of a sequence being computed and tokenized without any overlap between *k*-mers. Over-lapping *k*-mer tokenization, the *k*-mers of a sequence are computed and tokenized with a defined overlap. 7-10 Subword-Tokenization: WordPiece tokenization utilizes merge rules that prioritize merges based on a score calculated by dividing the frequency of the sub-word pair by the products of the frequencies of its component subwords. BPE tokenization (the example above is after one iteration) iteratively merges frequently occurring tokens until all pairs occur once (the example above could be merged again). SentencePiece uses either the BPE or Unigram algorithms to represent sequences as text as subword tokens.

The integration of deep learning into genomics has facilitated the development of powerful new ways to decipher biological sequences. The success of these models is not just a matter of architectural innovation; it is inextricably linked to the strategies used to tokenize the underlying sequences. This tokenization step involves converting raw strings of nucleotides into discrete units for computational processing. The evolution from simple motif-finding with CNNs to sophisticated sequence generation with LLMs has been a story of co-evolution, where new architectures demanded better tokenization, and new tokenization methods enabled more powerful models. This review traces this interdependent relationship, starting with foundational approaches and culminating in the state-of-the-art models that are pushing the boundaries of genomic prediction and understanding. We outline the deep learning architectures covered in this review, alongside any advantages or disadvantages, in Table 1, Table 3 respectively. We visually outline commonly used tokenization techniques for genomics in Fig. 1, and their advantages and disadvantages are further outlined in Table 2.

**Table 1:** Overview of deep learning models applied in genomics.

Model/Study Name	Architecture	Year	Context Length	Representation Method
DeepBind [2]	CNN	2015	101 bp	One-Hot Encoding
DeepSEA [82]	CNN	2015	1,000 bp	One-Hot Encoding
DANN [54]	DNN	2015	N/A	One-Hot Encoding
Basset [34]	CNN	2016	600 bp	One-Hot Encoding
DanQ [55]	CNN + biLSTM	2016	1,000 bp	One-Hot Encoding
DeepCpG [3]	Hybrid CNN/RNN	2017	1001 bp	One-Hot Encoding
ExPecto [81]	CNN + Aggregation	2018	40,000 bp	One-Hot Encoding
Basenji [33]	CNN with Dilated Convolutions	2018	131,000 bp	One-Hot Encoding
BPNet [5]	CNN with Dilated Convolutions + Residual Connections	2019	1,000 bp	One-Hot Encoding
DeepVirFinder [60]	CNN	2020	3,000 bp	k-mer Frequencies
ProtTrans [22]	Transformer	2020	2,048 aa	Amino Acid (Character-based)
ESM [28], [44], [62]	Transformer	2020	2,048 aa	Amino Acid (Character-based)
Enformer [4]	CNN + Transformer	2021	198,608 bp	One-Hot Encoding + CNN Downsampling
DNABERT [32]	Transformer (BERT)	2021	512 tokens	Overlapping k-mer
SpliceBERT [14]	Transformer (BERT)	2022	1,024 bp	Overlapping k-mer
ViBE [27]	Transformer (BERT-Hierarchical)	2022	512 tokens	Overlapping k-mer
DNABERT-2 [83]	Transformer (BERT)	2023	10,000 bp	BPE and SentencePiece
Geneformer [13], [71]	Transformer	2023	4,096 genes (V2)	Ranked Gene Expression
Nucleotide Transformer [19]	Transformer	2023	12,000 bp	Non-overlapping k-mer
ProGen [7], [46], [47]	Transformer	2023	8192 Tokens	Amino Acid
GenSLM [85]	Transformer (Hierarchical + Diffusion)	2023	131,072	Codon (Non-overlapping 3-mer)
HyenaDNA [50]	Hyena Operator (Convolutions + Element-Wise Gate Layers)	2023	1,000,000bp	Nucleotide-based
Mamba [26]	Selective State Space Model	2023	1,000,000 bp	Nucleotide-based
Evo [11], [49]	Transformer-Hybrid (Hyena + Attention)	2024	131,000	Nucleotide-based
Caduceus [66]	SSM (Bi-directional)	2024	131,000	Nucleotide-based
GENERator [77]	Transformer	2025	98,000	Non-overlapping k-mer
Lyra [58]	Hybrid (Gated CNN + SSM)	2025	65,535 bp	Nucleotide-based
Borzoi [45]	CNN + Self-Attention + U-net	2025	524,000 bp	One-Hot-Encoding

**Table 2:**
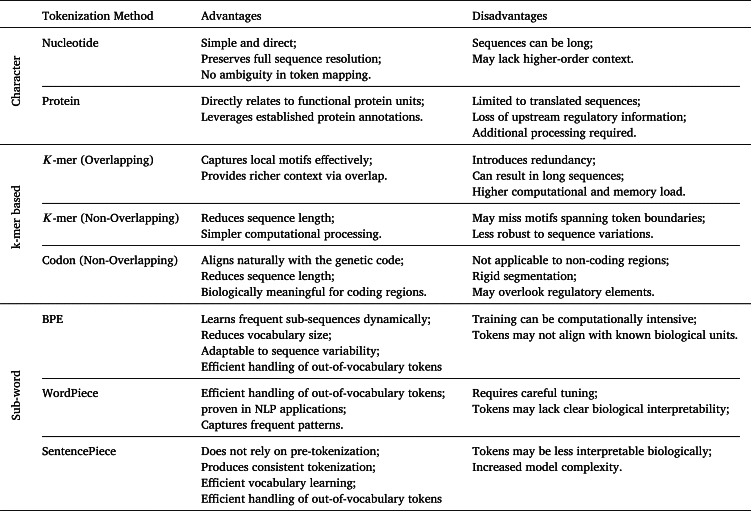
Tokenization methods applied within genomics alongside their advantages and disadvantages.

**Table 3:** Deep learning architectures used in genomics alongside their advantages and disadvantages.

Deep Learning Architecture	Advantages	Disadvantages
Convolutional Neural Networks (CNNs)	Efficient in capturing local patterns and motifs [37]; Parallelizable; Benefit from parameter sharing, reducing overfitting. [37], [41]	May struggle with long-range dependencies [8]; Performance is sensitive to kernel and pooling parameters [36].


Recurrent Neural Networks (RNNs)	Suited for sequential data; Capable of modeling temporal dependencies [65].	Prone to vanishing/exploding gradients [6]; Can be computationally intensive [75]; Limited capacity for more long-range dependencies [6].


Long Short-Term Memory (LSTM) Networks	Mitigate vanishing gradient issues [25], [29]; Can effectively capture long-range dependencies [25], [29];	More complex architecture [29]; Higher computational cost and development time [25], [29];


Transformer-based Architectures	Excellent at modeling long-range dependencies [20], [56], [72]; State-of-the-art performance in many sequence tasks [20], [23], [42], [72], [80].	High computational and memory demands; Require large training datasets [12]; Interpretability of attention weights can be challenging [31], [74].


Hybrid Architectures	Combine benefits of multiple approaches (e.g., CNN+Transformer) [4], [58]; Can capture both local features and global context [4].	Increased model complexity; May require careful regularization.

### 2.1 Foundations in genomics-based deep learning

Early applications of deep learning in genomics were dominated by CNNs. Models like DeepBind [2] and DeepSEA [82] pioneered the use of CNNs to identify motifs and predict protein binding sites directly from sequence data represented in a one-hot-encoded fashion. Similarly, other architectures such as the Deep Neural Network (DNN) used in DANN [54], which represented genetic variant information as a structured set of features for variant pathogenicity, and the CNN-based Basset [34], which utilized one-hot-encoding for functional activity classification, demonstrated the power of deep learning over traditional machine learning methods. Basenji expanded upon Basset, accepting longer one-hot-encoded sequences as input by performing dilated convolutions between vector sequences representing 128 base-pair regions to predict read coverage between sequences [33]. Dilated convolutions were also employed by BPNet [5], alongside residual connections, for base pair resolution prediction of transcription factor binding. Models based on CNNs tend to excel at local feature extraction and recognition of recurring patterns within genomic sequences due to their convolutional filters. These early models established the utility of CNNs for capturing local sequence features but were often limited in their ability to model long-range dependencies, a key challenge in genomics.

### 2.2 Pushing the boundaries of CNNs

In an attempt to model long-range dependencies more effectively, other deep learning model architectures have been explored in the field of genomics. DanQ is a hybrid model that integrates CNNs with bidirectional long- and short-term memory (biLSTM) networks [55]. Sequences were still one-hot-encoded, but by combining the local feature extraction capabilities of CNNs with the long-range dependency capture of recurrent networks, DanQ improved the prediction of function in DNA sequences, mainly where interactions occur over extended genomic distances. The recurrent connections present in DanQ allowed the model to significantly outperform models that used CNNs only, such as DeepSEA [55]. DeepCpG [3] is a model that can predict single-cell DNA methylation states using two modules: one that applies CNNs to extract features directly from the DNA sequence and another that uses recurrent networks to model dependencies between CpG sites within cells. Using this hybridized architecture, DeepCpG can be used to identify known and novel sequence motifs that predict the level or variability of DNA methylation [3]. Expecto [81] is another deep CNN-based model that also combines spatial transformations followed by linear models to predict, ab initio from DNA sequences, the tissue-specific transcriptional effect of mutations. Up to this point, these more complex model architectures were still working on a limited range of one-hot-encoded sequences. However, combining multiple models increases architectural complexity. The advent of the transformer architecture [72] provided an elegant solution for capturing long-range dependencies, leading to a wave of new genomic models that called for a change in sequence representation.

### 2.3 Transformer models and tokenization

One of the first cases involving the use of transformer architecture in genomics was performed by Clauwaert and Waegeman [17]. In this work, the authors show that transformer models, which are widely used in NLP tasks, could provide state-of-the-art results on whole genome sequence analysis and annotation. Using the self-attention mechanism, transformers can model dependencies across a more extended range in DNA sequences, enabling improved accuracy for these annotation and labeling tasks. Geneformer, a model composed of six transformer encoder blocks, utilized pretraining on 30 million single cell transcriptomes [71]. These transcriptomes of each single-cell were then given a rank value and presented to the model, essentially performing whole sequence tokenization of the transcriptome. Through pretraining, Geneformer's ability to predict dosage-sensitive gene variants was improved, lending support to the need for large-scale genomic databases for improved model performance and successful foundational model development [71].

Dalla-Torre et al. proposed the Nucleotide Transformer [19], this architecture utilized stacked transformer blocks of varying sizes and depth alongside a pretraining regimen on three separate datasets. They were able to apply the Nucleotide Transformer on several downstream tasks (Epigenetic Marks Prediction, Promoter Sequence Prediction, Enhancer Sequence Prediction, and Splice Site Prediction). The model utilized a *k*-mer tokenization technique where sequences of non-overlapping *k*-mers (k=6) were tokenized prior to the model, treating the DNA sequence as a sentence and the *k*-mers as words. In contrast to the Nucleotide Transformer architecture, the Enformer model leverages a hybrid setup; it first processes a one-hot encoded DNA sequence with convolutional layers that down-sample the input, effectively creating larger, abstract tokens. These tokens are then passed to transformer blocks to predict gene expression and enhancer-promoter interactions [4]. This method of representation allowed Enformer to outperform its predecessor, Basenji [4]. This hybrid approach was further evolved in the Borzoi [45] model, which builds upon the core Enformer architecture. Borzoi utilizes a stack of convolutional blocks for feature extraction, followed by self-attention layers to model long-range interactions across DNA sequences up to 524 kb in length. Finally, Borzoi employs a U-net architecture to increase the prediction resolution, enabling it to model RNA-seq coverage with high precision. As the size of the genomics-based tasks and datasets has increased, the use of LLMs for various downstream genomics-based tasks has seen increased research interests.

### 2.4 Expanding to large language models

The development of LLMs based on the transformer architecture has substantial improvements in various NLP applications, including machine translation [23], [72], sentiment analysis [20], and text summarization [42], [80]. Naturally, their adaptation to genomic-based tasks has been explored. One of the first transformer-based LLMs to be used in genomics, DNABERT [32] leveraged the BERT architecture [20] for various downstream tasks. Critically, it employed overlapping *k*-mer tokenization to represent DNA sequences, bridging NLP techniques with bioinformatics practices. Using this approach, DNABERT demonstrated that a BERT model, utilizing a masked language modeling (MLM) objective adapted for *k*-mer tokens during training, could effectively learn meaningful representations from genomic sequences. This self-supervised pretraining strategy allows the model to be subsequently fine-tuned with relatively smaller labeled datasets for various downstream tasks such as promoter prediction [32]. DNABERT's attention mechanism provides interpretability, highlighting specific sequence positions relevant to predictions. Inspired by DNABERT [32], ViBE [27] is a hierarchical BERT model that was developed explicitly for viral genome classification. Using overlapping 4-mer tokenization of single- or paired-end sequences, ViBE outperformed the latest alignment-free methods in all test cases included in the study [27].

Notably, the fixed k-mer tokenization strategy used in DNABERT [32] and ViBE [27] has key limitations. The vocabulary size grows exponentially with the value of *k*, creating a trade-off. Smaller values of *k* (e.g., 3) may fail to capture longer or more complex motifs, while large values of *k* (e.g., 11 or 13) can result in sparse vocabularies that increase computational demands. More importantly, this fixed vocabulary struggles with genetic diversity not contained in the development data. Any *k*-mer containing a rare variant or novel mutation not seen during training must be handled by a fallback strategy. A commonly used approach to handling out-of-vocabulary *k*-mers is the decomposition of the unknown *k*-mer into its individual base nucleotides, requiring the implementation of the decomposition logic and the inclusion of the base characters in the vocabulary. Another strategy is to include all possible sub-k-mers in the vocabulary from the start. However, this method can also lead to a combinatorially large vocabulary. In the worst-case scenario where no fallback is defined, the *k*-mer can be represented as a generic “unknown” token (“[UNK]”), effectively masking a potential meaningful signal. These challenges highlight some of the inflexibilities present with fixed *k*-mer tokenization schemes and the considerations that should be made when utilizing them. Addressing these inflexibilities represents a key frontier for genomic language models. A promising, yet underexplored, direction is the development of context-adaptive tokenization methods. Rather than relying on a fixed vocabulary, such methods could dynamically define token boundaries based on local sequence complexity.

To address the weaknesses of fixed k-mer vocabularies, the subsequent DNABERT-2 [83] replaced *k*-mer tokenization in favor of both Byte Pair Encoding (BPE) [68] and SentencePiece [39]. This change made DNABERT-2 [83] up to three times more efficient, improving performance in downstream tasks compared to its predecessor. The move of models like DNABERT-2 toward methods like BPE and SentencePiece [39] marks a critical shift away from fixed-size *k*-mers to data-driven tokenization strategies adapted from NLP. Unlike *k*-mer tokenization, which can lead to large, inflexible vocabularies, subword algorithms like BPE, WordPiece [67], and SentencePiece build a vocabulary directly from the training corpus. The core idea of these tokenizers is to build tokens by iteratively merging common character sequences (BPE) or by probabilistically segmenting sequences into the most likely sub-units (Unigram [38]). This creates a compact and efficient set of tokens that can represent common motifs as single units while breaking down rare sequences into smaller pieces. The scoring and loss functions for these widely adopted algorithms are summarized below:

(1) $$\text{BPE selects:}\;(x^{⁎},y^{⁎})=\arg\max_{\left(x,y\right)}\#(xy),\;s_{\text{BPE}}(x,y)=\#(xy),$$

(2) $$\text{WordPiece scores:}\;s_{\text{WP}}(x,y)=\frac{\#(xy)}{\#(x)\;\#(y)},$$

(3) $$\text{Unigram minimizes:}\;L_{\text{uni}}=-\sum_{w\in D}\log\sum_{t\in T(w)}\prod_{t\in t}p(t)\;,$$

where #(⋅) is the frequency in the corpus, T(w) is the set of all possible tokenizations for a sequence *w*, and p(t) is the unigram probability of a token. BPE's greedy approach in Equation (1) simply merges the most frequent adjacent pairs. In contrast, WordPiece [67] uses a likelihood score, as shown in Equation (2), to prioritize merges that are more statistically significant. The Unigram model, guided by the loss function in Equation (3), maintains multiple potential tokenizations for any given sequence, offering built-in regularization. The SentencePiece library [39] packages these BPE and Unigram models into a framework that operates directly on raw sequences, elegantly handling the end-to-end tokenization pipeline.

Other models have utilized large transformer-based architectures for tasks involving m-RNA or protein sequences. For instance, SpliceBert [14] was developed using pre-mRNA sequences for the prediction of sequence-based RNA splicing. ProtTrans [22] trained transformer-based models (ProtXL, ProtXLNet, ProtBERT, ProtAlbert, ProtElectra, and ProtT5) (Transformer-XL [18], XLNet [78], Albert [40], Electra [16], and T5 [57]) on massive protein sequence datasets (up to 393 billion amino acids) using self-supervised objectives like MLM. Similarly, EvolutionaryScale's ESM models [28], [44], [62], also pretrained on vast protein databases using MLM, have shown a remarkable ability to learn biologically relevant features, including aspects of 3D structure, directly from sequences. ProGen [46] is another large protein-based language model that uses a transformer decoder model trained on more than 280 million protein sequences in conjunction with conditional control tags to generate protein sequences. ProGen can generate plausible proteins, and its sequences have also been experimentally validated to show activities similar to representative sequences [46]. These models have mainly focused on the character-based tokenization of proteins, similar to nucleotide-based tokenization present in tasks involving DNA.

The application of these powerful sequence models extends broadly across bioinformatics, with comprehensive reviews detailing their use in proteomics and transcriptomics [59]. Furthermore, an emerging frontier is the development of multimodal models that directly integrate biological data, such as protein sequences, with natural language text such as clinical notes. This approach aims to create a more holistic understanding by combining the language of biology with human language, as demonstrated in recent work on text-protein foundation models [1], [21], [84].

Introducing a novel hierarchical architecture, GenSLM [85] tokenizes complete viral genomes using a codon-based tokenization method, akin to a non-overlapping k-mer tokenization, to capture long-range dependencies and generate viral sequences. GenSLM utilizes a top-level diffusion model to capture the global sequence context and a bottom-level transformer that captures the context at the codon level [85]. GenSLM was pretrained on a collection of various prokaryotic gene sequences to enable the model to learn broader biological knowledge; this pretrained model was then fine-tuned on SARS-CoV-2 full genomes. Another large foundational model, Evo [11], [49], leverages the striped hyena architecture [52] that interleaves deep convolutional operators for long-range signal processing, known as a hyena layer, with multihead attention layers utilizing rotary position embeddings [70]. This architectural adjustment has allowed for the use of nucleotide-based tokenization with the Evo models, maintaining full sequence resolution and permitting the model to extract local motifs over long distances.

Common training strategies for these genomic LLMs often involve large-scale self-supervised pretraining on vast unlabeled sequence corpora using objectives like masked language modeling or related techniques adapted from NLP, followed by fine-tuning on specific downstream tasks with labeled data. This pretrain/fine-tune paradigm has proven highly effective in leveraging large datasets while adapting models to specialized genomic problems.

LLMs and other transformer-based models are excellent at modeling long-range dependencies. These models leverage the self-attention mechanism to directly model interactions across sequences directly, explicitly allowing for the capture of functionally linked distant elements in genomic data. These architectures have also been shown to be scalable to large datasets, with many pretraining methodologies involving massive amounts of data, [11], [22], [28], [44], [49], [62], allowing these models to capture complex biological patterns that may be missed in smaller-scale methods. In addition, the self-attention mechanism helps add an additional layer to the interpretation and explainability of the model. Attention weights can be used to highlight specific tokens within a sequence that contribute the most to a given prediction, providing additional clues about regulatory mechanisms within genomic sequences. Although transformer architectures have seen wide success in NLP and genomics, they can require heavy computation and memory requirements, especially when processing longer sequences. These models are often much more complex, particularly when hierarchical or hybridization is used. Moreover, these models are often regarded as “data-hungry,” requiring massive and diverse datasets for practical training and generalization.

### 2.5 Emerging non-self-attention architectures

While transformer-based models have dominated recent deep learning research in genomics, the quadratic computational cost in relation to sequence length presents a significant challenge for analyzing entire chromosomes or vast genomic regions. This has spurred the development of alternative architectures that model long-range dependencies more efficiently. Among the first of these architectures to demonstrate state-of-the-art performance was HyenaDNA [50], a genomic foundation model explicitly designed to overcome the limitations of transformers. Instead of attention, HyenaDNA uses a Hyena operator, which is based on large, implicit convolutions parameterized by a small neural network. This design choice allows the model to scale sub-quadratically with sequence length, allowing HyenaDNA to process context lengths of up to 1 million tokens at a single-nucleotide resolution. Mamba [26] is another architecture that moves away from self-attention and uses a selective state space model (SSM) for efficient sequence modeling via a dynamic adaptation to input sequences and a hardware-aware design. An SSM maps a 1D input sequence to a latent state and then to an output. Mamba makes this process selective, allowing it to modulate its parameters based on the input context, enabling it to “forget” irrelevant information and “remember” relevant data over vast distances. This hardware-aware design, combined with bidirectional scanning in models like Caduceus [66], makes it exceptionally suited for modeling ultra-long DNA sequences without the computational burden of transformers. Both of these models efficiently handle long sequences and model long-range dependencies without utilizing a quadratic, $O(n^{2})$, attention mechanism. Lyra [58] is another model that does not use a self-attention mechanism, utilizing projected gated convolutions for local motif capture and diagonalized state-space models for global context aggregation. A key feature of these architectures is their approach to tokenization. Unlike transformers that often rely on k-mers or subword units to shorten sequences, models like Mamba and Caduceus typically operate directly on nucleotide-level tokens. The increased optimization of these architectures allows for them to handle longer inputs while remaining efficient. Therefore, the evolution of genomic deep learning is not merely a linear progression toward transformers but a broader exploration of specialized architectures tailored to the unique challenges posed by the structure and scale of biological data. While these models address some key limitations of transformer-based LLMs, a significant opportunity lies in designing architectures that directly integrate sequence representations with other data modalities. Future emerging architectures, or transformer-based LLMs, should also consider moving beyond unimodal approaches and leverage unified frameworks that fuse tokenized sequences with other data modalities.

## 3 Discussion

This review has surveyed the rapidly evolving landscape of tokenization techniques and deep learning architectures, particularly large language models (LLMs), applied to genomic sequence analysis. Although significant progress has been made in demonstrating state-of-the-art performance on various benchmarks, several key challenges and considerations emerge regarding the translation of these computational advances into robust and meaningful biological insights.

A primary challenge lies in optimizing sequence representation through tokenization. As reviewed, methods range from high-resolution nucleotide-level input, which preserves single-base detail but poses computational limitations and may obscure higher-order features, to various compression techniques like *k*-mers, adapted sub-word units (BPE, SentencePiece), and biologically motivated units like codons. These representations improve computational tractability and can implicitly capture some local context, but often at the cost of increased vocabulary size, potential loss of fine-grained information, and the introduction of token boundaries that may not align with biological function. Critically, beyond performance on downstream tasks, rigorously evaluating how well different tokenization schemes capture or obscure genuine biological signals (e.g., motifs, structural elements, reading frames) remains an open challenge requiring more sophisticated benchmarks.

Parallel advancements to deep learning architectures and training paradigms have driven progress in the development of highly accurate genomics-based tools. Architectures utilizing CNNs excel in local motif discovery, while RNNs/LSTMs offer sequential modeling capabilities. The subsequent adoption of transformer-based LLMs has been transformative, mainly due to the proficiency of the self-attention mechanism in modeling long-range dependencies. This capability is crucial for better understanding complex genomic regulation biologically, such as distal enhancer-promoter interactions or epigenetic modifications across large domains. The efficacy of these LLMs often hinges on large-scale, self-supervised pretraining on exceptionally large datasets of sequences, a strategy that leverages unlabeled data effectively but carries substantial computational costs. Alongside these costs, these models risk inheriting biases from the pretraining corpora. Furthermore, effectively fine-tuning these large pretrained models on specific, often smaller, labeled genomic datasets requires careful consideration to avoid overfitting and ensure generalizability on new, unseen data. The field needs continued critical evaluation to ensure that performance gains on benchmark tasks translate to reliable, generalizable biological discovery across diverse datasets and conditions.

Synthesizing these findings, several overarching challenges and future directions become apparent. First, computational efficiency remains paramount, especially as research moves toward analyzing longer sequences (e.g., entire genes with regulatory regions or whole chromosomes). This necessitates ongoing development of more efficient transformer variants (e.g., leveraging approaches such as the hyena layers used in Evo) and tokenization methods that compress sequences effectively without losing critical biological information. Second, bridging the gap between computational abstraction and biological meaning is vital. Future tokenization research should explore biologically-informed methods (beyond codons), context-adaptive tokenization, and robust frameworks for comparing their impact on model learning. Third, enhancing model interpretability and explainability is essential for building trust and extracting actionable insights. Moving beyond attention maps towards methods that provide causal explanations or link model internals directly to biological mechanisms (e.g., pathways, structures) is a key research frontier. Lastly, developing more robust evaluation strategies and benchmarks that assess generalization, calibration, and biological plausibility of model predictions is crucial to effectively steering the field.

Specific avenues for future work include systematically evaluating state-of-the-art models such as GenSLM [85] and Evo [11], [49] with diverse and dynamic tokenization strategies to precisely map the interaction between representation, architecture, and downstream performance. Continued exploration into sample-efficient training and domain adaptation techniques will also be vital for the application of large models in data-limited biological contexts. Ultimately, while the fusion of advanced tokenization and deep learning holds immense potential for genomics, significant research is still required to develop models that are not only predicatively accurate but also efficient, interpretable, and reliably grounded in biological reality.

## 4 Future work

Building on our exploration of tokenization techniques for genomic sequences, we identify three directions for an extension of previous work. Each direction is rooted in the idea that more genomic-focused tokenization techniques will drive better performance, clearer insights, and broader applicability of trained models.

### 4.1 Context-adaptive tokenization

A key limitation of the prevalent tokenization methods reviewed here, such as fixed length *k*-mers and codon-based tokenization, is their inability to capture variable-length functional sequence motifs. This review identifies a clear need for methods that can dynamically adapt token boundaries to the local sequence context in a biologically meaningful manner. Future research should seek to develop tokenization methods that adapt to the local sequence complexity via dynamic token boundaries. Using adaptive token boundaries, a more complete representation of motifs may be possible, resulting in a more comprehensive understanding of the underlying DNA sequence on behalf of the model. These methods may further explore the use of techniques such as reinforcement learning to accomplish this goal, potentially expanding the work performed in the MxDNA study [53]. A proposed method may be able to leverage a reward function that balances predictive accuracy with tokenization efficiency. Furthermore, the success of such an approach should be evaluated not only on the performance of the downstream tasks but also through a rigorous analysis of the learned vocabulary for enrichment of known biological motifs.

### 4.2 Tokenization-driven multimodal architectures

Genomic function arises from a complex interplay between the DNA sequence and its regulatory context. Additional insights may emerge when sequence data are integrated with orthogonal modalities such as epigenetic profiles, transcriptomic measurements, chromatin conformation data, or spatial information based on images. Future work should focus on developing unified embedding frameworks that jointly encode sequence tokens and auxiliary data within a shared latent space. Cross-attention and co-attention mechanisms are particularly promising for capturing dependencies between specific sequence features and their corresponding functional signals. The fusion of tokenized genomic sequences with other modalities has the potential to improve predictive performance on complex tasks such as annotation of regulatory elements, prediction of enhancer-promoter interactions, and prioritization of variant effects. It also offers opportunities for greater model interpretability by attributing outputs to combined sequence and regulatory features. Benchmarking these multimodal architectures across diverse datasets will be critical to evaluate their generalizability and to determine how integration of non-sequence data can resolve ambiguities that sequence-only models cannot. Finally, addressing scalability challenges through efficient tokenization, sparse attention, and modality-specific compression will be essential as these models are applied to increasingly large and heterogeneous datasets.

### 4.3 Scalable compression-aware tokenization

The shift towards long-read sequencing and whole-genome analyses presents a major scalability challenge for existing tokenization methods, which can produce prohibitively long input sequences when applied naïvely. This issue is particularly pronounced in repetitive or low-complexity regions of the genome, where standard tokenization schemes often fail to exploit redundancy effectively. As a result, there is increased pressure to develop both more efficient tokenization strategies and model architectures capable of handling extremely long and variable-length inputs. A critical research direction is the development of compression-aware tokenization schemes tailored to genomic data. Dictionary-based compression algorithms, such as LZW [73], LZ [30], Prefix-free parsing [10] and RE-Pair [35], provide promising avenues for reducing sequence lengths without sacrificing information content. These methods can collapse long repetitive regions into compact representations, producing a smaller number of tokens for highly redundant regions while preserving a rich token vocabulary for more variable and functionally important sequences. Furthermore, integrating such compression-aware schemes with hierarchical or multi-resolution modeling approaches could enable models to process genomic data at different levels of granularity focusing computational resources on informative regions while maintaining global sequence context. This could be particularly beneficial for large-scale tasks such as pangenome analysis, structural variant detection, and epigenomic profiling. To fully realize these benefits, future work should explore the design of hybrid tokenization pipelines that dynamically select between compressed and uncompressed representations based on local sequence complexity. In addition, evaluating the impact of such tokenization on downstream model interpretability and error propagation will be critical, especially for clinical applications where transparency is paramount. The development of benchmarks and standardized datasets for evaluating compression-aware tokenizers in genomics would also facilitate rigorous comparisons and accelerate progress in this area. Finally, coupling these methods with emerging architectures like state-space models or memory-efficient transformers may provide a scalable path forward for whole-genome deep learning.

## 5 Conclusion

We have surveyed the rapidly evolving landscape of deep learning architectures and tokenization strategies in genomics. The body of work discussed in our review highlights how traditional challenges in biological sequence analysis, such as extensive manual feature extraction, feature engineering, or the difficulty of capturing long-range dependencies, have spurred the development of innovative deep learning models. Architectures such as CNNs, RNNs, LSTMs, and LLMs have demonstrated significant potential to improve predictive accuracy and biological insight in several downstream genomic-based tasks.

A central feature of our review has been the critical role of tokenization in bridging the gap between raw genomic sequencing data and the deep learning models they are used to train. Effective tokenization not only facilitates the learning process by encoding complex sequencing information but also directly influences the interpretability and efficiency of the architecture used. We have surveyed a range of approaches, ranging from conventional *k*-mer-based methodologies to more dynamic techniques derived from NLP, and examined their respective advantages and limitations.

Despite the advancements covered, several challenges remain. The scalability of current models to handle increasingly large genomic datasets, the identification of more efficient biologically meaningful tokenization methods, and the need for more interpretable deep learning models are issues warranting further investigation. Addressing these challenges is essential as deep learning continues to be applied in genomics tasks and to translate computational insights into practical applications in medicine and beyond.

Future research should aim to develop more adaptive and robust tokenization strategies, integrate multi-model biological data, and enhance the interpretability of deep learning predictions. Such efforts will be key to advancing our understanding of genomic regulation and driving innovative solutions for biomedical research. This survey helps to lay the groundwork for these endeavors by summarizing current methodologies and identifying promising directions for future study.

Finally, choosing the appropriate model or tool for a given biological problem is critical. Key considerations include the size and quality of the available datasets, the need for model interpretability versus raw predictive performance, computational resource constraints, and the specific biological question at hand (e.g., motif discovery, variant effect prediction, or large-scale sequence classification). By matching these criteria to model characteristics, such as CNNs for local motif detection, RNNs/LSTMs for moderate-range dependencies, transformer-based LLMs for tasks requiring long-range context, or more recent non-self-attention-based models for their efficient performance over long sequences, researchers looking to implement these algorithms can make informed decisions and accelerate their path from data to discovery.

## CRediT authorship contribution statement

**Conrad Testagrose:** Writing – review & editing, Writing – original draft, Investigation. **Christina Boucher:** Writing – review & editing, Supervision, Project administration, Investigation, Funding acquisition.

## Declaration of Competing Interest

The authors declare the following financial interests/personal relationships which may be considered as potential competing interests: Christina Boucher reports financial support was provided by 10.13039/100000001National Science Foundation. If there are other authors, they declare that they have no known competing financial interests or personal relationships that could have appeared to influence the work reported in this paper.
